# Stop, look, listen: the need for philosophical phenomenological perspectives on auditory verbal hallucinations

**DOI:** 10.3389/fnhum.2013.00127

**Published:** 2013-04-09

**Authors:** Simon McCarthy-Jones, Joel Krueger, Frank Larøi, Matthew Broome, Charles Fernyhough

**Affiliations:** ^1^Department of Cognitive Science, ARC Centre of Excellence in Cognition and its Disorders, Macquarie UniversitySydney, NSW, Australia; ^2^Department of Psychology, Durham UniversityDurham, UK; ^3^Department of Philosophy, Durham UniversityDurham, UK; ^4^Department of Psychology, University of LiègeLiège, Belgium; ^5^Department of Psychosis Studies, King's College London, Institute of PsychiatryLondon, UK; ^6^Division of Mental Health and Wellbeing, Warwick Medical School, University of WarwickCoventry, UK

**Keywords:** hallucination, phenomenology, psychosis, schizophrenia, interdisciplinary

## Abstract

One of the leading cognitive models of auditory verbal hallucinations (AVHs) proposes such experiences result from a disturbance in the process by which inner speech is attributed to the self. Research in this area has, however, proceeded in the absence of thorough cognitive and phenomenological investigations of the nature of inner speech, against which AVHs are implicitly or explicitly defined. In this paper we begin by introducing philosophical phenomenology and highlighting its relevance to AVHs, before briefly examining the evolving literature on the relation between inner experiences and AVHs. We then argue for the need for philosophical phenomenology (*P*henomenology) and the traditional empirical methods of psychology for studying inner experience (*p*henomenology) to mutually inform each other to provide a richer and more nuanced picture of both inner experience and AVHs than either could on its own. A critical examination is undertaken of the leading model of AVHs derived from phenomenological philosophy, the ipseity disturbance model. From this we suggest issues that future work in this vein will need to consider, and examine how interdisciplinary methodologies may contribute to advances in our understanding of AVHs. Detailed suggestions are made for the direction and methodology of future work into AVHs, which we suggest should be undertaken in a context where phenomenology and physiology are both necessary, but neither sufficient.

## Introduction

The experience of hearing a voice in the absence of an appropriate external stimulus, formally termed an auditory verbal hallucination (AVH), is found in people with a range of psychiatric diagnoses, including schizophrenia, bipolar disorder, post-traumatic stress disorder, as well as in non-psychiatric populations (Larøi et al., 2012). In attempting to understand the causes of AVHs, researchers have explored the experience at multiple levels, including the neurological, cognitive, and sociological (McCarthy-Jones, 2012). Yet all these levels of explanation must take into account the findings of phenomenological studies of AVHs. A detailed knowledge of the phenomenology of AVHs is necessary, firstly, in order to ensure that models are explaining what voice-hearers are actually experiencing and, secondly, in order to obtain clues as to what may be underpinning the experience of AVHs (McCarthy-Jones et al., 2012).

Consistent with this proposal, Garcia-Montes et al. (2012) have argued that research should give people diagnosed with psychosis a “turn to speak,” i.e., that it should take a phenomenological approach, involving in-depth questioning of people about their subjective experiences, with questions that suspend or “bracket” presuppositions about the phenomena under investigation, including its normality or abnormality, and its causes (Stanghellini and Lysaker, 2007). Garcia-Montes et al. also suggest that giving people “increased importance in defining their experience may assist in fine-tuning concepts used by the cognitive tradition, usually taken from research in basic psychology … and extrapolated without further ado to the field of schizophrenia.” They note that this could lead to cognitive psychology “leaving behind its tendency to reduce symptoms or clinical phenomena to premade concepts.” Such an approach has the potential to interrogate some unexamined assumptions in the study of AVHs, even if they are eventually found to be innocent. For example, many models in the cognitive psychology paradigm assume that AVHs are altered forms of normal cognitive processes (such as inner speech or memory), and often uncritically recruit such concepts into their explanatory models. However, it is possible that AVHs may be a distinct experience, without roots in these normal cognitive processes, and thus requiring entirely new mechanisms and processes to explain them. One method by which such assumptions can be examined is through the use of philosophical phenomenology to undertake a detailed phenomenological investigation of AVHs.

Philosophical phenomenology is a systematic investigation of subjectivity, a consideration of experience from the first-person perspective of the “I” (Moran, 2000; Sokolowski, 2000). For phenomenologists, this first-person emphasis involves an analysis of basic structures of consciousness such as intentionality, self-awareness, temporality, embodiment, spatiality, agency, and intersubjectivity. It is through these basic structures that the world becomes manifest within our conscious life. Phenomenology is thus concerned with the constitutive processes that give our experience of the world and ourselves its formal coherence. In light of this first-person orientation, phenomenology might be thought of as the foundational science for psychopathology (Fuchs, 2010; although, as we noted above, there is no necessary link between AVHs and states that could be described as pathological).

Methodologically, phenomenological investigations of consciousness involve a disciplined *seeing* (Gallagher, 2012). This means that phenomenology (literally, the “science of appearances”) looks to provide a rigorous description of experiential phenomena as they reveal themselves to consciousness. As Heidegger puts it, this disciplined seeing involves letting “that which shows itself be seen from itself in the very way in which it shows itself from itself” (Heidegger, 1962, 58/34).

The first step of phenomenological seeing begins by suspending taken-for-granted assumptions or judgments about the cause, normality, or reality of what is experienced (Broome et al., 2012). This formal suspension (or *epoché*, as Husserl, 1960, termed it) of metaphysical and scientific judgment is a port of entry into the inner structure of experience. For, rather than focus on external factors or explanations, we instead carefully observe what we experience just as we experience it; our subsequent descriptions are based on this “naïve” presuppositionless seeing. Within the clinical context, this means that the investigator suspends etiological and diagnostic considerations and instead re-focuses on the character and meaning of the patient's experience from their perspective. Questions here include: what is it like for the patient to have a particular experience or be in a particular mental state (e.g., to be depressed or hear voices)? How do salient phenomena within this experience manifest to the patient? What is the meaning of this experience for the patient and how does it relate to their present situation?

In relation to the experience of AVHs, employing a philosophical phenomenological approach and being guided by the experience itself may result in the deprioritization of aspects of their phenomenology which, for a range of good or bad reasons, have previously been deemed salient (although, to be clear, this approach does not set out to depriortize any aspect of the experience stressed by contemporary approaches). For example, as a result of historical factors certain aspects of AVHs have become of a priori concern to the mind sciences, such as the quasi-fetishized property of their location, i.e., whether AVHs are perceived as being internally or externally located to the head. Similarly, widely used AVH assessment tools, such as the Psychotic Rating Scales (PSYRATS-AH: Haddock et al., 1999) have been influential in regard to which aspects of AVHs are deemed important to enquire about (such as loudness, duration, frequency), without any evidence that these are either the aspects research should be focusing on, or those which voice-hearers themselves think are important. We may also consider the very use of the term “voice-hearing” which may not accurately capture what the person is experiencing (McCarthy-Jones, 2012), and instead suspend such ways of seeing to allow the person to report salient aspects of the experience to them, in the phrasing that they prefer.

The second step involves imaginative variation, the eidetic reduction, a process by which we intuit the essence (*eidos*) of the phenomenon under consideration; we use our imagination to alter various aspects of the phenomenon in order to discern the invariant features that define it as the sort of entity that it is. For example, when one sees a particular apple, one can imagine that it might be a different color, texture, size, or weight, or have a different flavor, without it thereby becoming something other than an apple. None of these features are part of its essence. A similar process can be applied to anomalous experiences ranging from affective depersonalization in melancholic depression to disruptions of *ipseity* and embodiment within schizophrenia/AVHs (see below). The payoff of this process is that the phenomenologist gets a clearer picture of the phenomenon's prototypical features, information which is then used to generate more nuanced descriptions of the patient's overall experience. These descriptions are further specified by subjecting them to intersubjective scrutiny within a community of fellow scientists, phenomenologists, and voice-hearers themselves (categories which are, of course, not mutually exclusive). In this way phenomenology can recreate the experiential dimension of psychiatric disorders and in so doing provide acute descriptions of anomalous phenomena (Parnas and Zahavi, 2000).

In relation to the experience of AVH, though, such a process may become problematic due to the heterogeneity of the phenomenology of the experience which makes the boundaries of AVHs hard to define (McCarthy-Jones, 2012). For example, experiences which are traditionally classed under the umbrella term “AVH” may range, phenomenologically, from an experience which is just like hearing another person speak, to an experience that is more thought-like than voice-like, through to an experience of a message being communicated to oneself that takes the form of a soundless, silent “voice” (Moritz and Larøi, 2012; McCarthy-Jones, 2012). Philosophical phenomenology may hence be able to examine whether these are qualitatively distinct experiences (involving distinct neural circuitry) or if they lie on a continuum with each other—e.g., a core experience which may range on dimensions such as its acoustics from clearly heard words to silent messages.

The third step is to return to the experience itself in order to assess the descriptive adequacy of these descriptions and categories. Within psychopathology, this involves a return to the clinical context. Here the phenomenologist can check the appropriateness of her/his findings by the phenomena she/he encounters (Fuchs, 2002). This process of recreating and analyzing the patient's lived experience thus takes the phenomenologist down into the basic structures of consciousness. It provides valuable insight into how these structures organize non-pathological experiences of self and world and, crucially, how and where these basic structures become compromised or disrupted within anomalous experience.

Whilst philosophical phenomenology is likely to benefit our understandings of a range of experiences, AVHs appear to be an arena where it can be particularly fruitful. This is because there has been both a failure to understand the background of “ordinary” inner experiences that AVHs are (implicitly or explicitly) defined against, as well as a failure to understand exactly what the experience of having AVHs is like. Before continuing though, it is worth being explicit as to what philosophical phenomenology can and cannot offer AVH research. It is not the aim of philosophical phenomenology to offer an explanation of the mechanistic processes underpinning AVHs, which is a task for neurocognitive approaches, but instead to facilitate this indirectly through providing a more accurate description of the experience. This can be of use in a number of ways, some of which we have already discussed. However, two key related benefits are that it can, (1) provide an account of the experience that can be the explanatory target of neurocognitive models of AVHs, and (2) allow the evaluation of existing neurocognitive models against this experience, i.e., if existing models are not explaining the actual experience of AVHs, but rather an incomplete, misleading, or partial portrait of the experience, then they are flawed and in need of revision. Despite this, some argue (e.g., Merleau-Ponty, 1962), that there can be no truly presuppositionless seeing, no “total” epoché, and that, just like cognitive psychology, philosophical phenomenology has its own set of concepts that influence the aspects of AVHs that are attended to, such as the embodiment of the experience or its temporal or spatial character. Such insights have been gained via phenomenological reflection on the nature of experience. However, techniques such as the epoché can at least significantly improve our descriptions of phenomena, bracketing our assumptions re: causation and the “natural attitude” even if they are still viewed through some form of interpretive lens.

## Inner experience and AVHs

Although there are many models of AVHs, such as memory-based models (Waters et al., 2006a), hypervigilance models (Dodgson and Gordon, 2009), and social deafferentation models (Hoffman, 2007), we will focus in this paper on how philosophical phenomenology may be applied to what is currently the dominant model (in terms of being the most empirically investigated, as well as most discussed) model of AVHs, the inner speech model. This model proposes that AVHs result from a disturbance to the process whereby inner speech is attributed to the self (e.g., Frith, 1992; Leudar et al., 1997). However, progress in this area has been hampered by a lack of attention to the phenomenological properties of inner speech and, relatedly, AVHs. In particular, there is a need for improved empirical research on key properties of inner speech that have been proposed to be of value in explaining the relation between inner speech and AVHs (Fernyhough, 2004; Jones and Fernyhough, 2007).

One such factor is *dialogicality*, which refers to the ability of inner speech to incorporate multiple perspectives on reality (Fernyhough, 1996, 2004). Dialogicality has been proposed to result from the development of inner speech through the internalization of structured linguistic exchanges with caregivers and others during the course of development (Vygotsky, 1934/1987; Fernyhough, 1996). This quality of inner speech has been proposed as an explanation for why AVHs often manifest voices of others alongside that of the self (Fernyhough, 2004; Jones and Fernyhough, 2007). A second important quality is *condensation*, the tendency of utterances in inner speech sometimes to appear phenomenally as having a condensed or “note-form” quality. Condensation has been proposed as a further reason why inner speech can take multiple forms, such as in the proposed distinction (Fernyhough, 2004) between “condensed inner speech” (in which the internal utterance is fully stripped-down and abbreviated) and “expanded inner speech” (in which internal utterances retain their full linguistic structure). Transition between forms of inner speech (condensed and expanded) has been put forward, in an extension of the basic inner speech model of AVHs, to explain why voices can suddenly intrude into consciousness (Fernyhough, 2004).

The empirical study of inner speech has of course been hampered by its unobservability. Some support for the dialogicality and condensation dimensions of inner speech was provided by McCarthy-Jones and Fernyhough (2011) who, using a self-report scale (the Varieties of Inner Speech Questionnaire), found the existence of these dimensions in a healthy sample of adults. Other empirical methods proving useful in the study of inner speech are dual-task methods in which the language system is temporarily blocked by, for example, articulatory suppression, and experience sampling methods such as the phenomenological Descriptive Experience Sampling (DES; Hurlburt and Heavey, 2006).

There has been little research on the phenomenological properties of inner speech and AVHs in those who hear voices. In the only study to provide a systematic analysis of inner speech phenomenology alongside AVH phenomenology, Langdon et al. (2009) found no significant differences in inner speech phenomenology between people diagnosed with schizophrenia who heard voices and non voice-hearing healthy controls. For example, there were no differences between the two groups in the form, speed, and pragmatics of their inner speech, and no relations between patients' inner speech and their voices in terms of variables such as frequency and pragmatics. However, there was a trend toward reduced dialogicality of inner speech in the patient group (in the sense of inner speech being less likely to take on an overt dialogic form), potentially suggesting a relation between a reduced normal inner dialogue and the presence of AVHs.

Of course, inner speech only forms a subset of inner experiences (Hurlburt and Heavey, 2006), and a recent study of the phenomenology of AVHs has suggested that a diverse range of inner experiences, such as verbal and non-verbal memories, as well as inner speech, may form the basis of AVHs. McCarthy-Jones et al. (2012) assessed the phenomenology of AHs in 199 psychiatric patients, using an interviewer-led semi-structured interview. In addition to reporting on a comprehensive range of properties of AHs, this study also employed cluster analysis (clustering by variable and hence identifying within-individual differences, rather than between-individual differences) and found four subtypes of AH, which they termed Constant Commanding and Commenting AVHs, Own Thought AVHs (which did not address the voice-hearer, spoke in the first-person, were experienced as being similar to memories, and possibly being one's own voice/thoughts), Replay AVHs (which were reported as being identical to previously experienced heard speech), and Non-verbal AHs (which were either language which did not make sense, or non-verbal sounds). It appears plausible that each of these subtypes of AH may result from distortions to distinct forms of inner experience, and that a better understanding of normal inner experience is therefore required before this can be mapped onto the phenomenology of AHs.

## “*P*henomenology” and “*p*henomenology”

The empirical methods of psychology for studying inner speech and other forms of inner experience such as AVHs entail clear limitations. Retrospective introspection of the form demanded by self-report studies is likely to be unreliable in certain circumstances, while dual-task methods gain little traction on phenomenology and rely on potentially misplaced assumptions about the recruitment of inner speech in cognitive tasks. Although DES is founded in phenomenological principles and is careful to ensure the bracketing of presuppositions, it can be criticized for its failure to generate generalizable empirical data. Philosophical phenomenology, which we denote here as *P*henomenology (phenomenology with a big “P”), can potentially enrich the methods of self-report, introspection, etc. that psychologists and cognitive scientists have traditionally relied on, which we refer to as *p*henomenology (phenomenology with a small “p”).

*P*henomenology can supplement and enrich *p*henomenology because the former works at a distinct but nevertheless complementary level of analysis to the latter. That is, whereas *p*henomenology and its methods of self-report, introspection, etc. yield important data about the specific *contents* of experience (i.e., *what* a subject is experiencing), *P*henomenology is—in addition to this data—also concerned with the formal *structures* of experience (i.e., *how* the subject is experiencing the “what”). This “transcendental” aspiration is essential to *P*henomenological methodology (Husserl, 1989).

Again, this transcendental aspiration is apparent in *P*henomenology's concern with how basic structures inherent in consciousness (e.g., intentionality, self-awareness, temporality, embodiment, spatiality, agency, intersubjectivity, etc.) organize and constitute experience and imbue it with a first-personal character. Data from *p*henomenological reports (e.g., patient vignettes in psychiatry) can lend important clues to how and where these basic structures become compromised or disrupted within anomalous experience. However, *P*henomenology can further contextualize these often fragmentary or isolated reports within a broader transcendental context. This is because *P*henomenology offers a sophisticated framework for describing experience and existence that enables the psychopathologist to address concrete issues of diagnosis and treatment while remaining mindful of how these local concerns relate to overarching issues such as time, space, self, and intersubjectivity (Parnas and Zahavi, 2000). Accordingly, *P*henomenology does not simply consider symptoms in isolation (i.e., as localized manifestations of brain dysfunction); nor does it reduce diagnostic entities to statistically relevant clusters of symptoms (Fuchs, 2010). Rather, these are considered in the broader context of the subject and the whole of consciousness in which they emerge, that is, as typical modes of human experience and existence through which the subject constitutes her experience of self, world, and other.

As Thomas Fuchs (2010) notes, this structural emphasis of *P*henomenology is a search for what he terms “psychopathological organizers” connecting single features (e.g., affective depersonalization in melancholic depression or autism in schizophrenia) within a larger experiential gestalt. This emphasis “helps define mental disorders on the basis of their structural experiential features, linking apparently disconnected phenomena together” (Fuchs, 2010, p. 549). The end result—in light of these complementary levels of analysis—is that *p*henomenology and *P*henomenology can together provide a richer and more nuanced picture of the phenomenon under consideration than can either approach on its own.

## Phenomenological philosophy and AVHs

### The ipseity model of AVHs

One attempt to utilize phenomenological philosophy to help understand AVHs has come from Sass and Parnas (2003). An examination of their resultant account of AVHs is informative of the strengths and limitations of the application of phenomenological philosophy to AVHs. Their model involves a phenomenological analysis of ordinary experience, as well as that experienced by people with AVHs, and then the use of this analysis to propose what processes may be underpinning AVHs. Sass and Parnas's approach is derived from a phenomenological analysis of two facets of the intentional act: (1) a pre-reflective embeddedness in the world, and (2) a tacit or pre-reflective self-awareness or ipseity (literally, “self” or “itself”). The term “ipseity” refers to the experiential sense of being a subject of experience, i.e., one's own first-person perspective on the world. The basic sense of ipseity in normal consciousness, argues Sass (2003), is reflected in someone “whose experiences are unified and owned rather than merely flying about loose” (p. 244).

Sass and Parnas (2003) argue that there are occasions, such as in schizophrenia, where this basic sense of self or ipseity becomes fragmented or otherwise disturbed. In the case of schizophrenia, disturbed ipseity exhibits two main features. The first is hyper-reflexivity, a form of exaggerated self-consciousness in “which something normally tacit becomes focal and explicit” (p. 430). For example, some patients report that normally tacit sensorimotor processes animating everyday behavior (e.g., getting dressed, drinking coffee, interacting with others, etc.) may lose their automaticity. Instead, the background repertoire of proprioceptive and kinaesthetic processes informing this behavior move to the foreground of the patient's focal attention; they become hyper-aware of the effort required to produce each gesture or movement—so much so that their body is eventually experienced as a mechanical object, resulting in an experience of disembodiment or “self-alienation” (p. 429). Alternatively, other patients report that particular details of a scene, or specific qualities of faces or persons, stand out with a kind of hypersalience; they are dislodged from the gestalt of the situational context and thus appear strange or uncanny (Wiggins and Schwartz, 2007). Even the perceptual act itself may rise to the level of focal awareness (e.g., “I became aware of my eye watching an object,” Stanghellini, 2004, p. 113). Hyper-reflexivity thus objectifies normally tacit, pre-reflective processes of agency and perception.

In a later paper (Nelson et al., 2009), the authors make clear that although hyper-reflexivity is a concept that includes an exaggerated intellectual or reflective process, it is not “at its core, an intellectual, volitional, or ‘reflective’ kind of self-consciousness. It primarily refers to acts of awareness that are automatic (non-volitional) and not intellectual in nature, as in the case of kinaesthetic experiences ‘popping’ into awareness” (Nelson et al., 2009, p. 809). As hyper-reflexivity makes focal what was once tacit, the experience can hence not be transparently inhabited by the self; hyper-reflexivity introduces a rupture within the basic structure of experience. This leads to the second, complementary component of ipseity disturbance: a diminishment of self-affection, which Sass and Parnas (2003) define as a reduction in the sense of basic self-presence; “the implicit sense of existing as a vital and self-possessed subject of awareness” (p. 429). For example, patients may report feeling an inner distance from their stream of consciousness (“I saw everything I did like a film-cameram” Sass, 1992, p.132), or “an inner void” or “lack of inner nucleus” where the self would normally be (Parnas and Handest, 2003). As ipseity disturbances, hyper-reflexivity and diminished self-affection thus erode the basis sense of self-presence and perspectival coherence that enables us to maintain an experiential grip on the world and on ourselves as embedded in the world.

Sass and Parnas (2003) propose that AVHs (and schizophrenia more generally) result from such an ipseity disturbance. They argue that in texts such as Ey (1973), Tissot (1984), Naudin et al. (2000), an altered state of self-awareness can be seen to occur before AVHs. Specifically, they argue that “the patient experiences his or her own subjectivity as becoming in a certain way ready for something strange to happen … Mental processes and inner speech … are no longer permeated with the sense of selfhood but have become more like introspected objects, with increasingly reified, spatialized, and externalized qualities” (p. 432). Commonly encountered AVHs, such as voices commenting on a hearer's on-going behavior, are, in Sass and Parnas' view, “emblematic of the self-consciousness that generates this self-alienation.” From this they conclude that AVHs “involve a sense of alienation from and a bringing to-explicit-awareness of the processes of consciousness itself.” Sass argues that this occurs through “an automatic popping-up or popping-out of phenomena and processes that would usually remain in the tacit background of awareness” (Sass, 2003, p. 156). As such they “do not involve the addition of anything new but only an awareness of what is always present (e.g., of inner speech, the perfectly normal medium of much of our thinking) in the context of diminished self-presence” (Sass and Parnas, 2003, p. 433). In their view, AVHs therefore “represent the perfectly normal phenomena of ordinary human experience—which, however, are radically transformed because of being lived in the abnormal condition of hyper-reflexive awareness and diminished self-affection” (p. 433).

### Limitations of the ipseity account

#### Phenomenological fusion

This interesting account has a number of notable limitations. Firstly, and most problematically for an ostensibly phenomenological account, although this model is strong in its analysis of the phenomenology of disturbances to normal experience (e.g., ipseity disturbances) and how this may be applied to AVHs, it lacks a comprehensive understanding of the phenomenology of AVHs to which is it trying to link. It therefore demonstrates what could be termed a lack of “phenomenological fusion,” i.e., a failure to link the known phenomenology of inner experience to the known phenomenology of AVHs. This criticism has previously been noted by Leudar and Thomas (2000) who state that Sass' “characterization of voices of schizophrenics [sic] is based on case materials which reflect traditional construals of voices in psychiatry rather than on the ground-floor experiences of individuals with schizophrenia” (p. 95). For example, Sass (1992) states that “the voices schizophrenics [sic] hear tend to emanate not from any particular person or object in external space but from inside the body or from the sky” (p. 233), and that patients with schizophrenia most frequently have voices which “have more of a conceptual or cognitive than a sensory or perceptual taint, as if heard with the mind rather than the ear” (p. 233). Neither of these observations is consistent with the observed phenomenology of the majority of AVHs (Nayani and David, 1996; Moritz and Larøi, 2008; McCarthy-Jones et al., 2012). Indeed, Sass' latter observation above comes from a statement of Bleuer in which Bleuler only says voices “may” take this form. However, Sass, at the time of this theory's development, did not have access to the large systematic studies of AVHs available today (e.g., Nayani and David, 1996; McCarthy-Jones et al., 2012), and hence the recourse to less systematized studies of AVH phenomenology is understandable. The lesson we may take from this is that the philosophical phenomenological approach needs to be applied both to normal experience and to AVHs, and to linking these together.

#### Specificity to AVHs

A second limitation is that despite the apparent argument made for a causal role of ipseity in AVHs, in a later paper Sass and colleagues (Nelson et al., 2009) argue that “ipseity disturbance seems to be independent of symptom manifestation,” still being present in the remitted phase of schizophrenia, hence making it a “trait or underlying marker of vulnerability, independent of the expression of this vulnerability in the form of psychotic symptoms” (p. 809). Similarly, Garcia-Montes et al. (2012) observe that high levels of “self-focused attentions are not exclusive in patients with auditory hallucinations, but that, in general, they characterize all patients with positive psychotic symptoms.” This could be interpreted as the proponents of this theory arguing that ipseity disturbances are a necessary, although not sufficient cause of AVHs. However, Sass focusses on AVHs in people diagnosed with schizophrenia, which is not only a contested diagnostic entity (Boyle, 2002) but a diagnosis that only contains around a third of people who hear voices (McCarthy-Jones, 2012). It could therefore potentially be the case that ipseity disturbances are linked to schizophrenia *per se* and have no causal relation with AVHs. It is also possible that ipseity disturbances are not found in other populations who hear voices, and are therefore not necessary for AVHs, and even if ipseity disturbances were found to be a necessary but not sufficient cause for AVHs, this would still leave the question as to what other additional factors are required for AVHs to ensue. Finally, it is also possible that in some populations or situations ipseity disturbances may be sufficient for AVHs. For example, the presence of AVHs in people undertaking intense introspection, such as the Desert Fathers (Christian monks in the third century who retired to the deserts of Egypt to pray: McCarthy-Jones, 2012) is at least suggestive that self-focus might be a sufficient cause of some AVHs.

#### Empirical testing and levels of explanation

At present there is very limited empirical evidence supporting Sass's ipseity account of AVHs. Although Sass (2003) was originally explicit that his account was “largely descriptive or interpretative rather than explanatory in nature” (p. 244), a more recent paper by Sass and colleagues (Nelson et al., 2009) has gone on to make some specific hypotheses, such as that the ipseity model predicts “an increase in self-focusing as causing a tendency to experience the object of focus as other-than-self (i.e., externalising or objectifying self-experience)” (p. 813). Linking this to a neurological level of explanation, they further state that this account “would predict that psychotic phenomena should be associated with increased cortical midline system (CMS) activity, to the extent that the disturbances of hyper-reflexivity and diminished self-affection suggest an increase in self-focusing as causing a tendency to experience the object of focus as other-than-self” (p. 813). Although phenomenological work leading to predictions at a neurological level is a promising way forward (see below), unfortunately for this specific hypothesis, activation of such structures during AVHs was not found in a recent meta-analysis (Jardri et al., 2011).

A recent paper by Garcia-Montes et al. (2012) considers the relation between Sass and Parnas's (2003) work and contemporary cognitive psychology. Garcia-Montes et al.'s overall argument is that there are noticeable parallels between “hyper-reflexivity” and some cognitive models of schizophrenia/AVHs that concentrate on attentional processes in such patients. However, it is unclear quite how Sass and Parnas's (2003) concept of hyper-reflexivity maps onto established psychological constructs. One possibility is that it relates to the psychological concept of meta-cognition (Garcia-Montes et al., 2012), which includes a range of items, including cognitive self-consciousness. However, a recent meta-analysis of the association between of meta-cognition and hallucination-proneness found only a weak association (Varese and Bentall, 2011). Nevertheless, what can be seen from this is that phenomenological philosophy needs to engage with (and potentially extend or revise), existing psychological constructs, in order to operationalize and test hypotheses that phenomenological philosophy has generated.

A further empirical limitation of this account is that other theories of AVHs predict the exact opposite to Sass and Parnas (2003). For example, Dodgson and Gordon (2009) argue that hypervigilance AVHs result specifically when attention is externally focussed. There is hence the need for an empirical investigation into the locus of attention of voice-hearers immediately preceding AVHs, which should be a priority for future experience-sampling studies of this phenomenon.

#### Benefits in terms of informing neurocognitive research

Although there is the need for phenomenological philosophy to engage with the concepts of existing neurocognitive work, it is also worth considering how it may extend these paradigms through critique. For example, Sass and Parnas's (2003) model can be considered in relation to the source-monitoring account of AVHs. Source-monitoring accounts of AVHs argue that a deficit in the skill of being able to distinguish between self-generated internal cognitions and non self-generated external perceptions leads the former to be mistaken for the latter, resulting in AVHs (Bentall, 1990). “Source monitoring” is used as a global term to cover both reality monitoring (the ability to differentiate between internally generated cognitions and external perceptions) and self-monitoring (the ability to differentiate between self- and other-produced stimuli). Although Sass and Parnas (2003) state that their account is “rather different” (p. 432) to the established self-monitoring deficit account of AVHs, they do not clearly set out their points of difference (in a later paper, they state that self-monitoring accounts are “redolent” of their ipseity model; Nelson et al., 2009).

In either case, Nelson et al. (2009) note that source-monitoring studies often require a reflective judgement about the source of a stimulus, making it unclear whether conscious self-reflection or the pre-reflective processes emphasized in their phenomenological accounts of ipseity disturbance in schizophrenia are being assessed. This inconsistency in experimental tasks may be able to account for the limitations in the existing psychological source-monitoring literature, which is somewhat contradictory. For example, whilst a recent meta-analysis has suggested specificity of source-monitoring deficits to AVHs (Waters et al., 2011), other studies have instead suggested that it is delusional ideation, not AVHs, that is linked to source-monitoring deficits (Allen et al., 2006; see McCarthy-Jones, 2012, for a review of the current evidence). There is hence the need for further consideration as to whether source-monitoring tasks involve conscious self-reflection or pre-reflective processes, and to examine these two separate forms of source-monitoring in relation to AVHs specifically, to see if both, neither or just one of these is related to them.

#### Summary

In summary, accounts of AVHs drawn from phenomenological philosophy may be valuable but need to ensure that: (1) if building from the phenomenology of normal inner experience, that this is then mapped onto the actual phenomenology of AVHs, (2) if descriptions of the experience are developed into a mechanistic account of AVHs, that they offer a mechanism specific to AVHs and not to schizophrenia *per se*, and (3) that their proposals lead to empirically falsifiable predictions at both a neural and cognitive level, clearly operationalizing their concepts in the language of these disciplines through collaboration with colleagues in such areas. For example, if a philosophical phenomenological approach were to predict that memory disturbances play a causal role in AVHs, then it would need to go on to predict how this may be reflected in neural activation during AVHs, what differences in performance on standard cognitive tests of memory would be expected, and what new memory tasks may potentially be needed in order to detect predicted changes. The resultant benefits of this approach are that it may both offer us a better understanding of the phenomenology of AVHs, and offer valuable critiques of existing psychological constructs, such as source-monitoring.

Once such original first-person data has been collected, it may then be utilized within a wider interdisciplinary research program, to guide the discovery of new objective (third-person) data at the neurophysiological level. Such an approach is already advocated in the methodology of neurophenomenology (Varela, 1996), in which trained introspection leads to first-person data which can then guide investigation at the third-person, neurophysiological level. Neurophenomenological work in non-AVH related fields (Lutz et al., 2002) can be seen to suggest how AVHs may be explored using neurophenomenology. For example, the use of *P*henomenology to detect distinct aspects of the hearing voices experience, such as the claim noted above that an altered state of self-awareness occurs before AVHs, could then be used to lead a search for whether these states have distinct neurological components, and how AVHs result from a cascade of neural activity resulting from an altered state of self-awareness.

Another example of a wider interdisciplinary research program engaging with the phenomenology of hearing voices comes from the work of the “Hearing the Voice” project at Durham University (e.g., Macnaughton, 2011). In this project, work on both the *P*henomenology and *p*henomenology of the hearing voice experience is being undertaken from disciplines including modern and medieval literary studies, theology, philosophy, psychology, and the medical humanities, with each discipline attempting to offer unique insights into the *P*/*p*henomenology of the voice-hearing experience (for example, medieval literary studies providing insights into how hearing voices was experienced and understood in this period of history through analysis of texts and documents from this era). Previous work has already demonstrated how, for example, historical analyses can offer us insights into the hearing voices experience (Jones, 2010). The Hearing the Voice project is further engaging with cognitive neuroscientific perspectives to examine how phenomenology and neuroscience may be mutually informative, leading to better understandings of the voice-hearing experience, and new ways to help people who are distressed by such experiences.

Such methodologies also allow that, in addition to first-person data informing work at the third-person level, this process may also work in reverse (e.g., through the neurophenomenological principle of mutual constraints), with third-person neurological findings informing the study of the first-person phenomenology of the experience (Varela, 1996). An approximation of this process can be drawn from some recent studies. For example, Diederen et al.'s (2010) functional magnetic resonance imaging study of the areas of the brain activated immediately before AVHs showed involvement of the parahippocampal gyrus, a region of the brain implicated in memory processes, suggesting that memory processes may play a role in the aetiology of AVHs. This, in part, motivated a later phenomenological examination of the involvement of memory in AVHs (albeit, using *p*henomenology rather than *P*henomenology) by McCarthy-Jones et al. (2012) who found that a notable subset of people with AVHs (39%) reported that their AVHs were identical and/or similar to “replays” of memories of things people had previously heard. Further work along these lines, in which first-person (phenomenological) and third-person (neurophysiological) perspectives mutually inform each other, is hence to be encouraged.

## Conclusions and empirical priorities for further progress in this area

Phenomenological philosophy clearly has a role to play in creating a better understanding of AVHs. However, this appears likely to necessitate a multiple stage process. First, there is the need to use phenomenological philosophy to better understand the *P*henomenology of ordinary inner experience, as well as the potential variability in properties of inner experience of specific relevance to AVHs, such as the tendency for inner speech in non voice-hearers to take on a perceptual nature. Second, as we noted above, there is the need to assess whether unusual forms of ordinary inner experience form the raw material of AVHs, or whether AVHs are unrelated to such processes and are qualitatively different, new forms of experiences. *P*henomenological study of both ordinary inner experience and AVHs should contribute to addressing this question.

Third, despite the recent large scale study (McCarthy-Jones et al., 2012) of the *p*henomenology of AHs noted above, there is still the need for *P*henomenological work that explores AHs using the techniques of phenomenological philosophy. For example, McCarthy-Jones et al. found that 12% of patients reported that their AVHs were identical to memories of previous things that had been spoken to them, and that 31% said their AVHs were similar to memories. Yet phenomenological philosophy methods are likely to be needed to probe the *P*henomenology of these experiences, and to establish what exactly participants meant by relating their voices to memories, and the characteristics of these experiences that led them to be labeled AVHs, as opposed to simply intrusive memories (cf. Waters et al., 2006b). Similarly, careful *P*henomenological analysis of the varieties of inner speech and AVHs will benefit our understanding of any relations between these experiences. Such work will need to ensure that it creates what we have termed phenomenological fusion, mapping through the phenomenology of inner experience onto the phenomenology of AVHs.

Fourth, there is the need to use phenomenological philosophy and other methods derived from humanities disciplines to assess the meaning of AVHs for the person hearing them and examine how this relates to their present situation. Such findings are likely to prove of benefit for the development of cognitive behavioral techniques (CBT) aimed at relieving voice-hearers' distress, particularly with regard to being able to create meaningful formulations. Phenomenological philosophy is also likely to provide other benefits to CBT therapists. For example, the greater degree of presuppositionless seeing offered by the technique of epoche, may help limit the degree to which pre-existing theories of AVHs, whether these be trauma-based, developmental insult-based, or recreational drug triggered-based accounts, are employed in the therapeutic context, allowing the unique personal circumstances of the patient to guide the development of their formulation which is open to multiple explanations at first. Any such studies would benefit from having a longitudinal methodology, in order to create a developmental profile of how individuals move from normal inner experience to AVHs (e.g., Raballo and Larøi, 2011), and whether this is a gradual shift or a sharp change.

Finally, there is the need to build on such studies to map new first-person data onto their underpinning neurophysiological mechanisms (and vice versa), with these approaches reciprocally informing and mutually enriching the other. In this sense, the design of future studies will need to undertake what Gallagher (2003) has termed phenomenological front-loading.

One of the strengths of phenomenological philosophy, its suspension of presuppositions about the causes of the phenomena under investigation, is also a limitation for researchers interested in the causes of AVHs. There is hence the need to explain the results of phenomenological philosophical investigation. If, for example, there is a disturbance of ipseity, what may cause this? It may be important to try to understand why a person enters such a state. This may result from a wide range of situations, such as intense emotion/stress driving the person to turn his/her attentional resources inwards, social isolation resulting in attention turning inwards, or consciously intended meditative introspection, as with mystics throughout the centuries. Similarly, it needs to be established why the “automatic popping-up or popping-out of phenomena … that would usually remain in the tacit background of awareness” (Sass, 2003, p. 156) occurs? Why is there this increased awareness of “what is always present (e.g., inner speech)” (Sass and Parnas, 2003, p. 433)? When does it occur and why? What are the neural correlates of this process? Furthermore, which aspects of the person's inner life are the focus of the person's attention? Does this process account for both increased self-focus on auditory mentation (resulting in AVHs) as well as visual imagery (resulting in visual hallucinations)? It should also be considered that alternative mechanisms may cause different types of AVHs. For example, hyper-reflexivity fits well with AVHs in many healthy individuals who experience them under conditions of intensive introspection. However, it appears less clear how this maps onto AVHs of people who may have their attention externally focused during the AVH (Dodgson and Gordon, 2009).

A final consideration is how *p*henomenology and *P*henomenology may work together to improve our understanding of AVHs. That is, how can philosophical phenomenology potentially enrich the methods of self-report, introspection, etc. that psychologists and cognitive scientists have traditionally relied on? In this sense there needs to be a dialogue between research methods based on the principles of philosophical phenomenology and the standard semi-structured interview which is the mainstay of qualitative research.

In conclusion, phenomenological philosophy is likely to be able to make a significant contribution to our understanding of AVHs, and to be a profitable and necessary partner for neurophysiological research. Phenomenology and physiology are both necessary, but neither are sufficient.

### Conflict of interest statement

The authors declare that the research was conducted in the absence of any commercial or financial relationships that could be construed as a potential conflict of interest.
